# Supplementary figures and images for: Phosphorylation of luminal region of the SUN-domain protein Mps3 promotes nuclear envelope localization during meiosis
Source: eLife. 2021 Sep 29;10:e63119. doi: 10.7554/eLife.63119 (PMC8570693; doi:10.7554/eLife.63119)

Figure 3C Rao et. al.

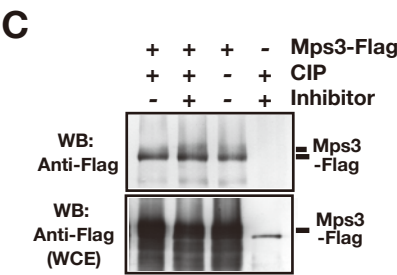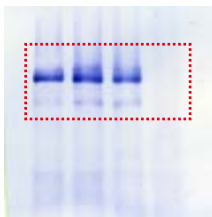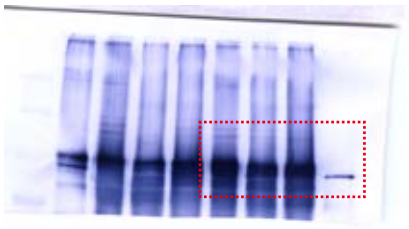

Supplement: Figure 3—source data 2. [file elife-63119-fig3-data2.zip › Mps3-FIg-source/Mps3-Fig.3C-source.pdf]

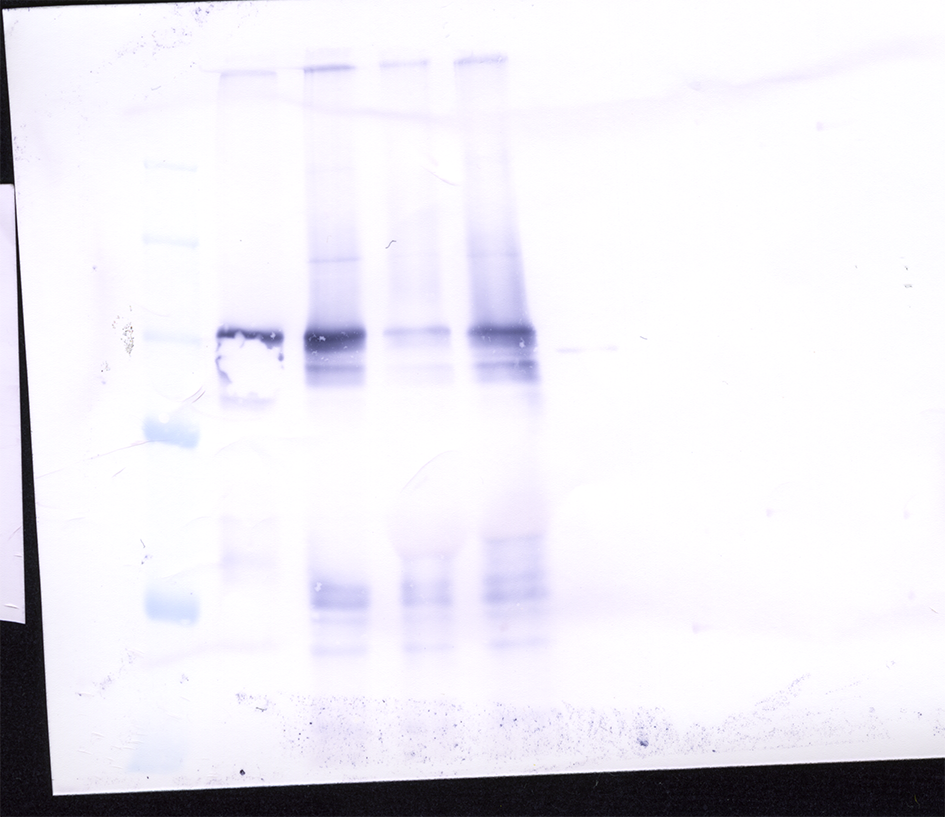

Supplement: Figure 3—source data 2. [file elife-63119-fig3-data2.zip › Mps3-FIg-source/Fig.3Dtop.tif]

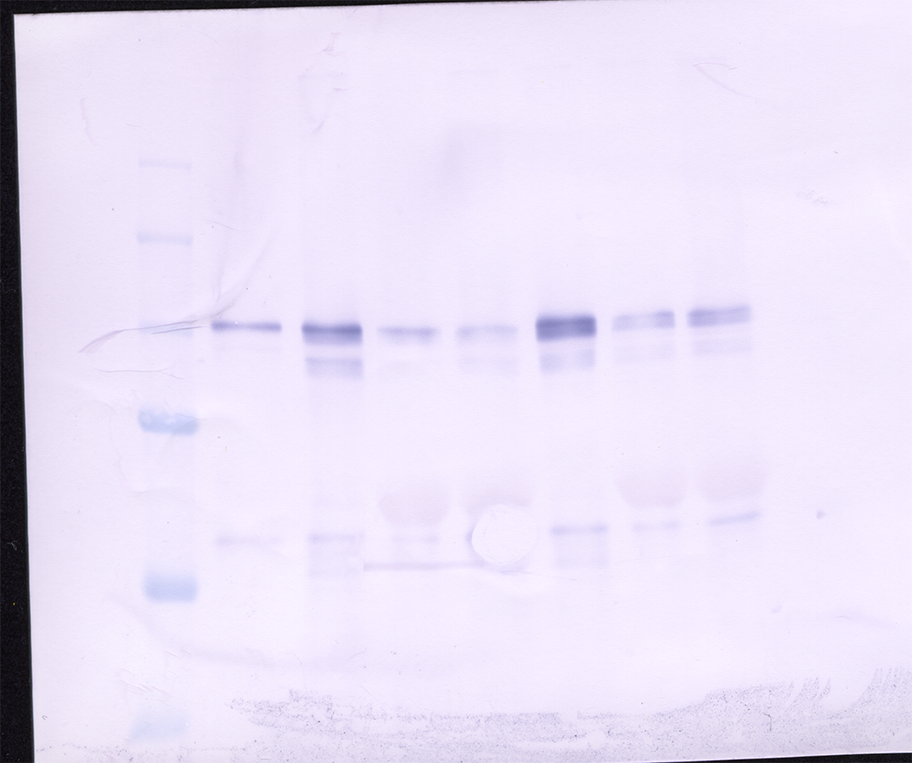

Supplement: Figure 3—source data 2. [file elife-63119-fig3-data2.zip › Mps3-FIg-source/Fig.3Htop.tif]

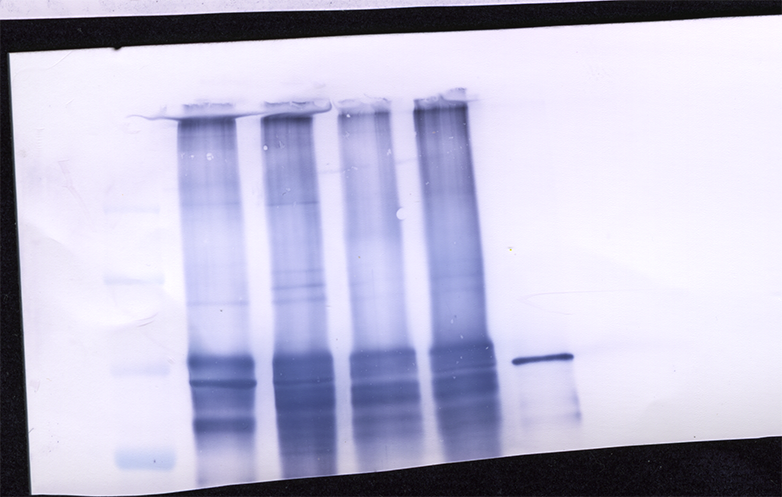

Supplement: Figure 3—source data 2. [file elife-63119-fig3-data2.zip › Mps3-FIg-source/Fig.3D-bottom.tif]

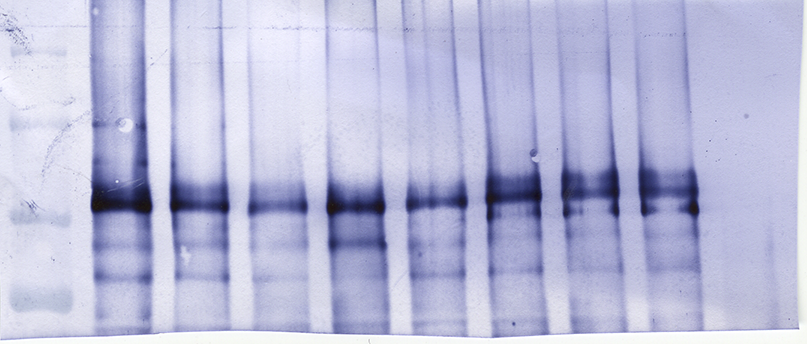

Supplement: Figure 3—source data 2. [file elife-63119-fig3-data2.zip › Mps3-FIg-source/Fig.3I-top.tif]

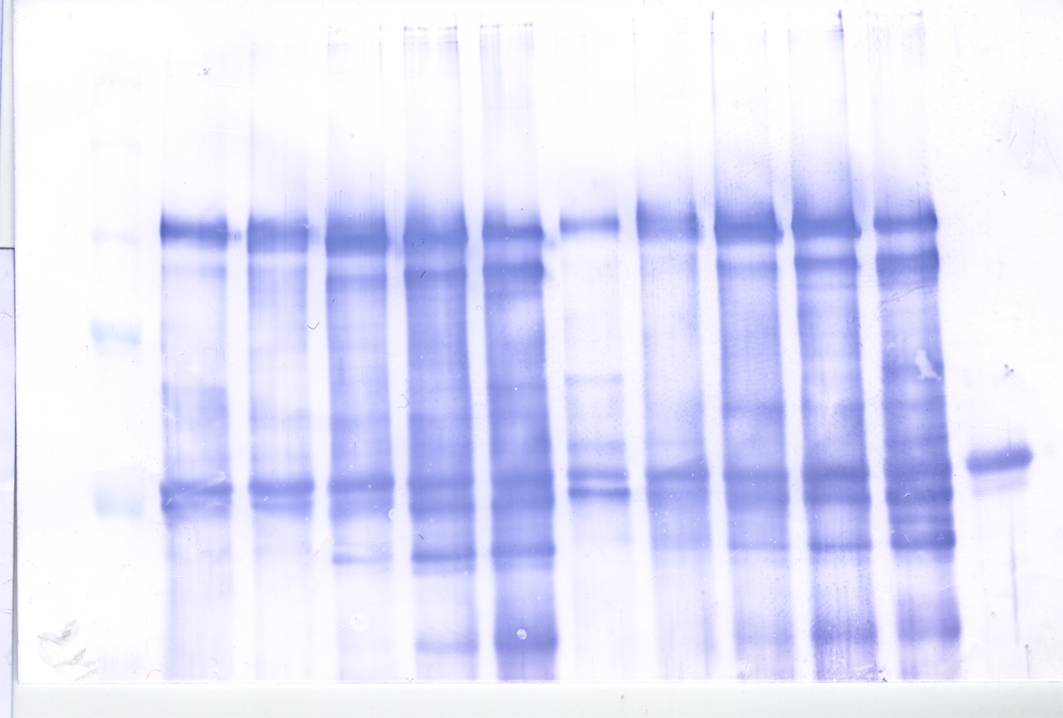

Supplement: Figure 3—source data 2. [file elife-63119-fig3-data2.zip › Mps3-FIg-source/Fig.3C-left-top.tif]

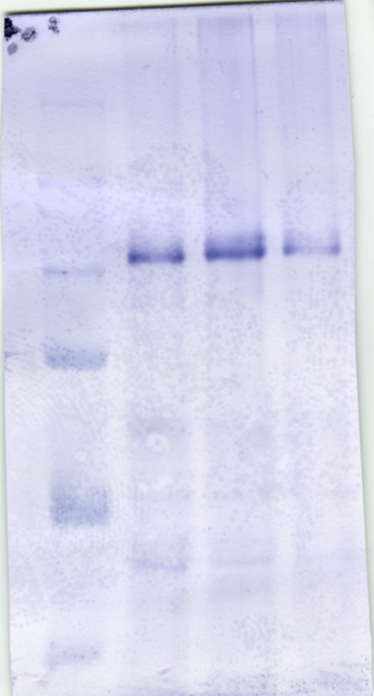

Supplement: Figure 3—source data 2. [file elife-63119-fig3-data2.zip › Mps3-FIg-source/Fig.S1top.tif]

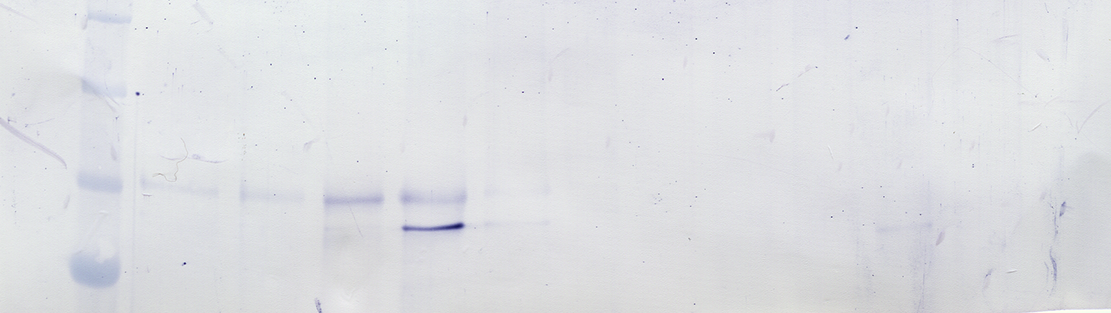

Supplement: Figure 3—source data 2. [file elife-63119-fig3-data2.zip › Mps3-FIg-source/Fig.3I-bottom.tif]

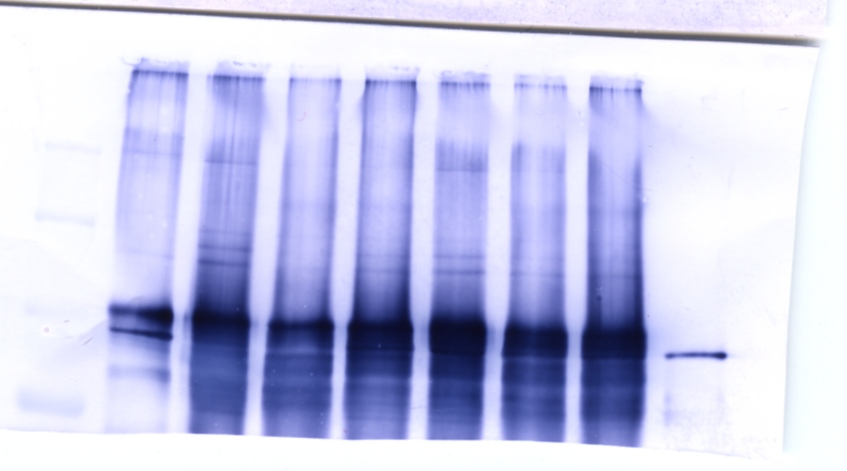

Supplement: Figure 3—source data 2. [file elife-63119-fig3-data2.zip › Mps3-FIg-source/Fig.3Bbottom.tif]

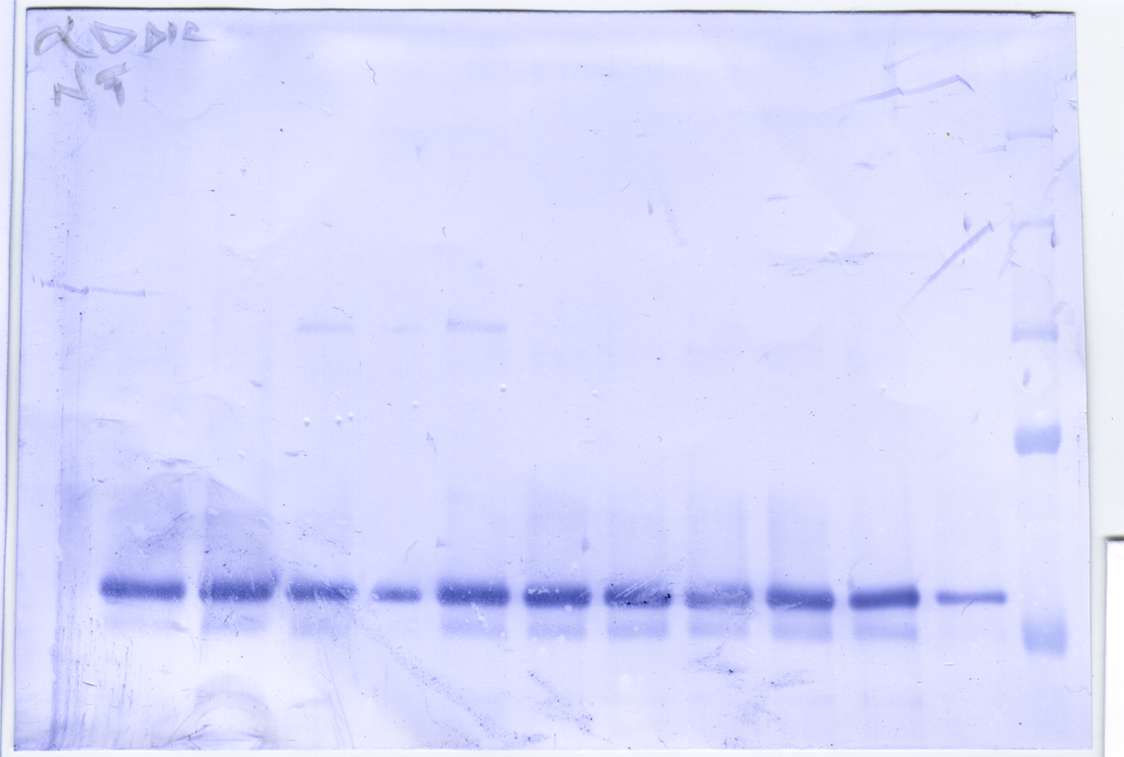

Supplement: Figure 3—source data 2. [file elife-63119-fig3-data2.zip › Mps3-FIg-source/Fig.3C-right-bottom.tif]

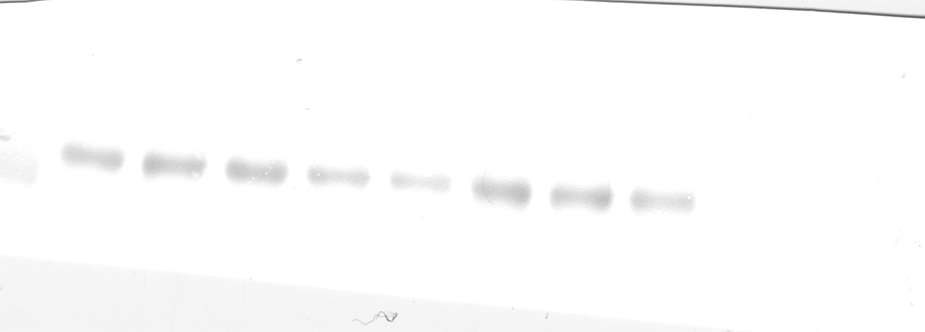

Supplement: Figure 3—source data 2. [file elife-63119-fig3-data2.zip › Mps3-FIg-source/Fig.3G-tub.tif]

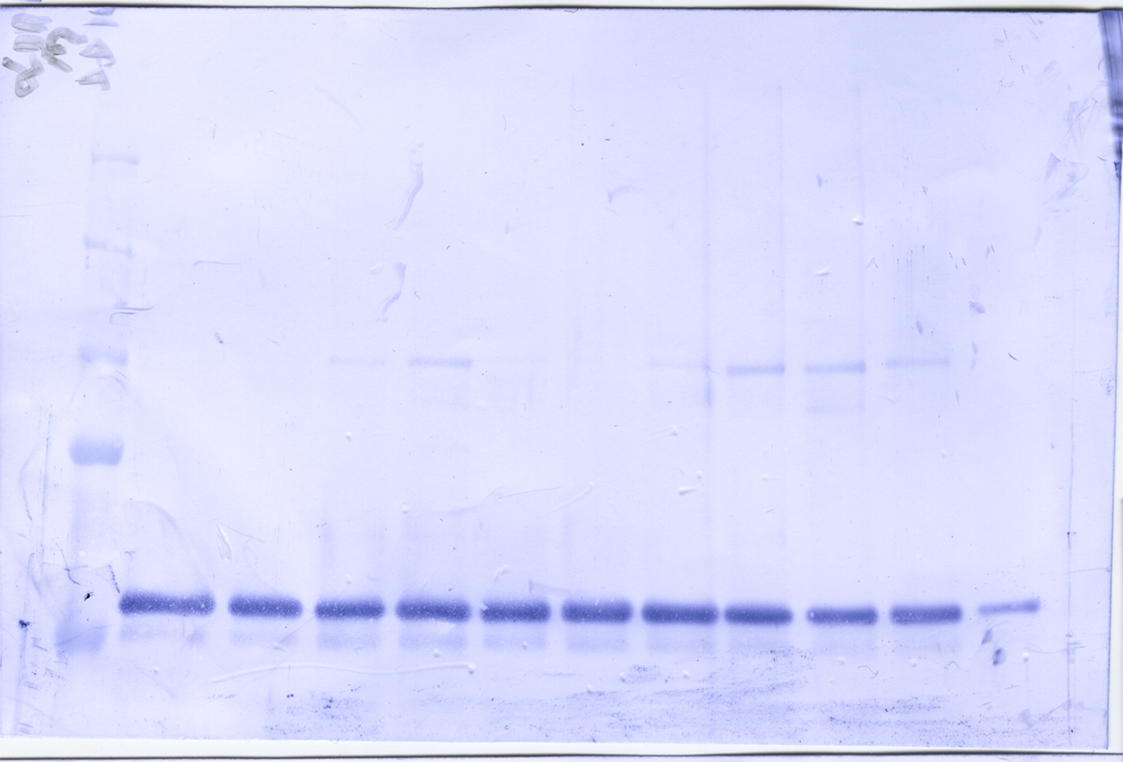

Supplement: Figure 3—source data 2. [file elife-63119-fig3-data2.zip › Mps3-FIg-source/Fig.3C-WT-bottom.tif]

Figure 3I Rao et. al.

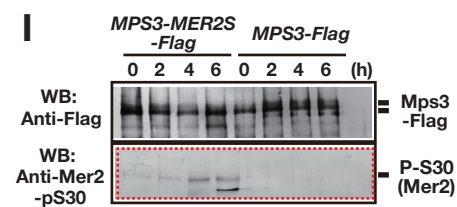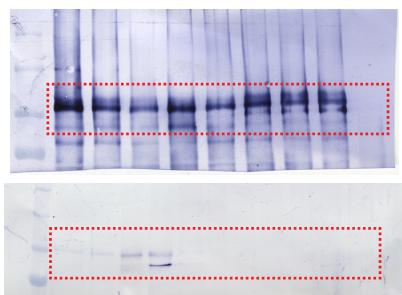

Supplement: Figure 3—source data 2. [file elife-63119-fig3-data2.zip › Mps3-FIg-source/Mps3-Fig.3I-source.pdf]

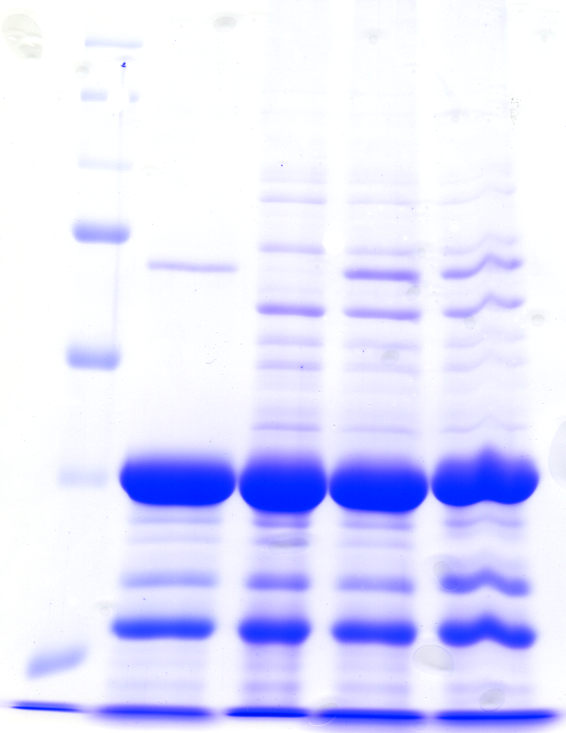

Supplement: Figure 3—source data 2. [file elife-63119-fig3-data2.zip › Mps3-FIg-source/Fig.3F-CBB.tif]

Figure 3D Rao et. al.

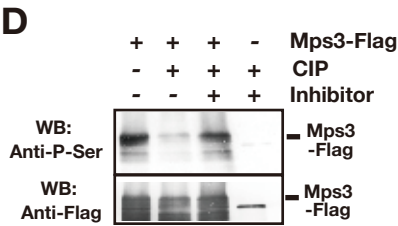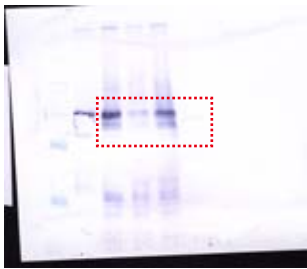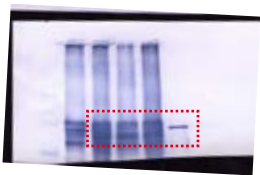

Supplement: Figure 3—source data 2. [file elife-63119-fig3-data2.zip › Mps3-FIg-source/Mps3-Fig.3D-source.pdf]

Figure 3G Rao et. al.

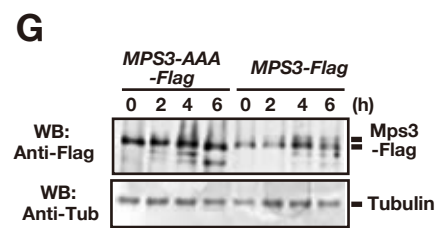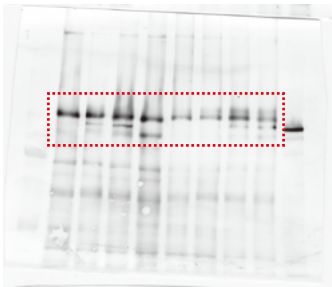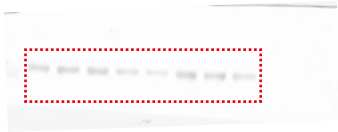

Supplement: Figure 3—source data 2. [file elife-63119-fig3-data2.zip › Mps3-FIg-source/Mps3-Fig.3G-source.pdf]

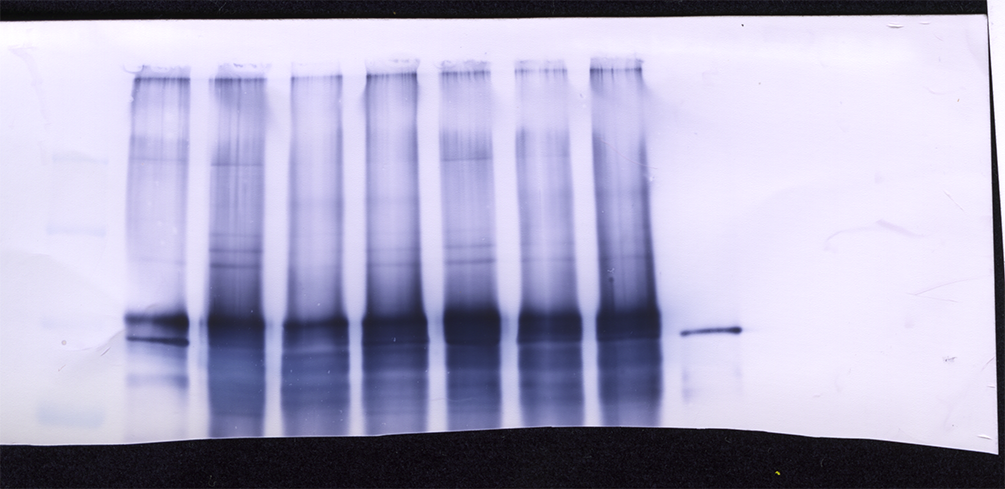

Supplement: Figure 3—source data 2. [file elife-63119-fig3-data2.zip › Mps3-FIg-source/Fig.3H-bottom.tif]

Figure 3C Rao et. al.

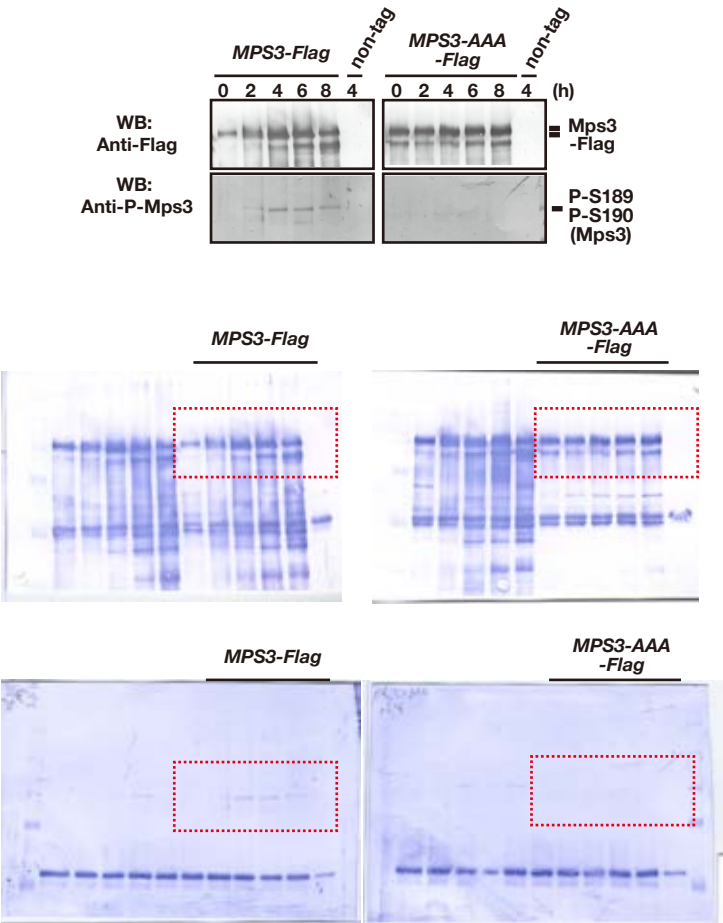

Supplement: Figure 3—source data 2. [file elife-63119-fig3-data2.zip › Mps3-FIg-source/Mps3-Fig.3B-source.pdf]

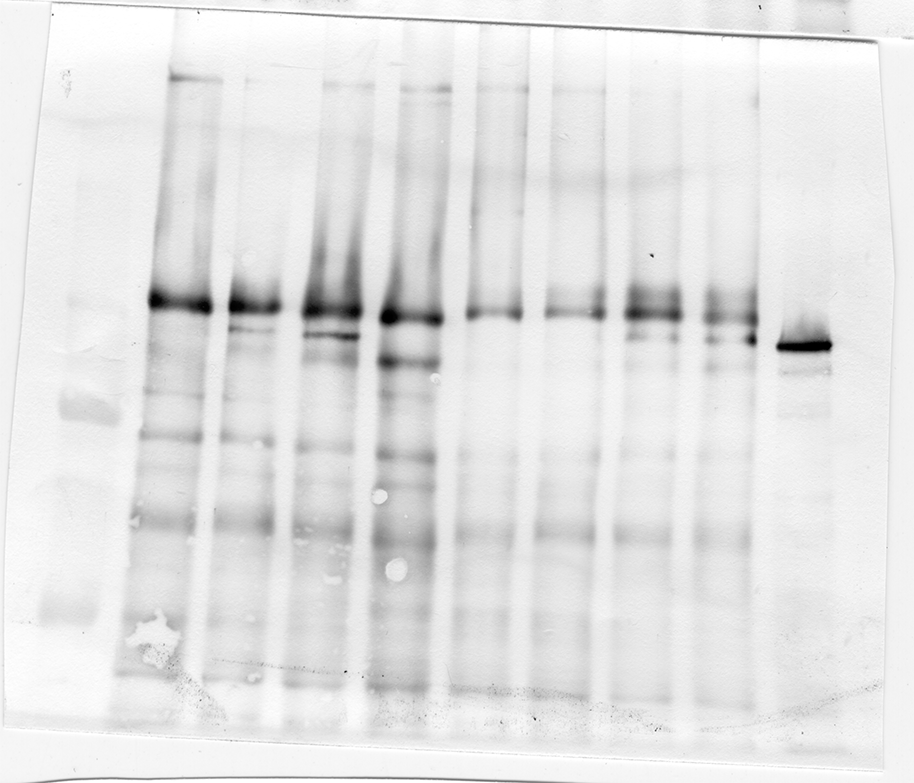

Supplement: Figure 3—source data 2. [file elife-63119-fig3-data2.zip › Mps3-FIg-source/Fig.3G-top.tif]

Figure 3H Rao et. al.

H

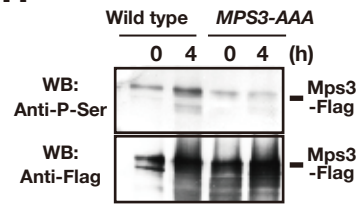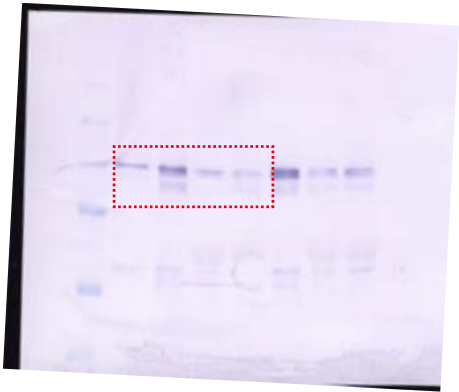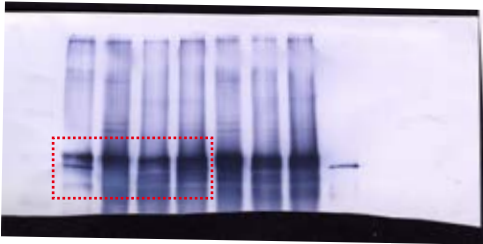

Supplement: Figure 3—source data 2. [file elife-63119-fig3-data2.zip › Mps3-FIg-source/Mps3-Fig.3H-source.pdf]

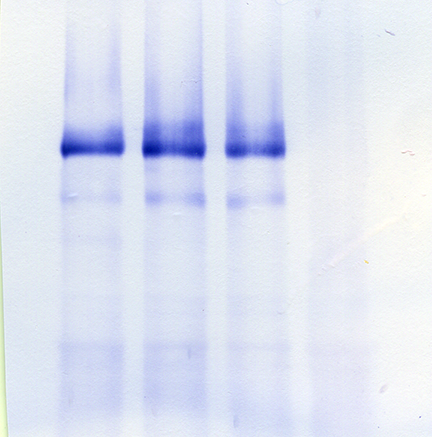

Supplement: Figure 3—source data 2. [file elife-63119-fig3-data2.zip › Mps3-FIg-source/Fig.3Btop2.tif]

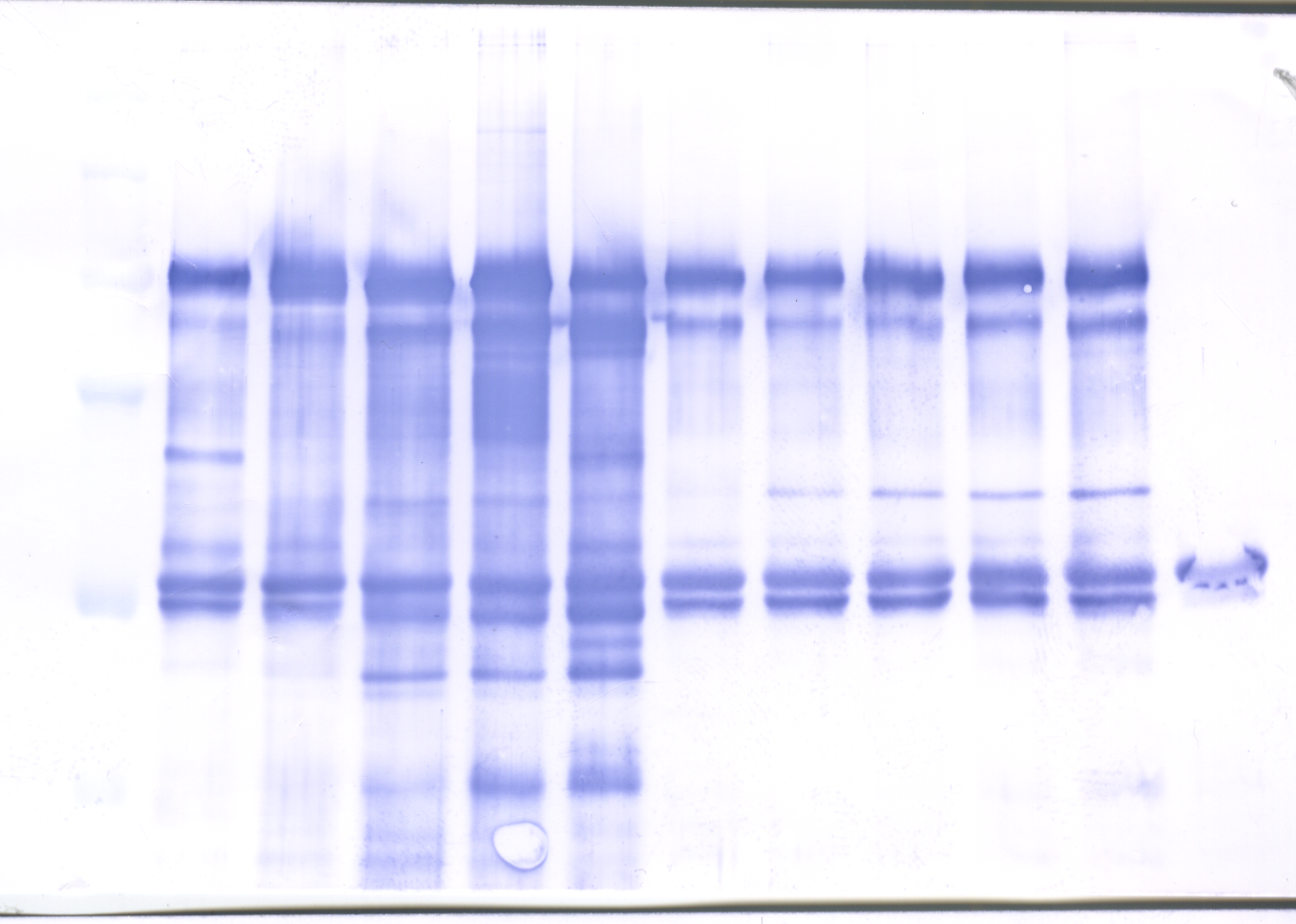

Supplement: Figure 3—source data 2. [file elife-63119-fig3-data2.zip › Mps3-FIg-source/Fig.3C-right-top.tif]
