# Supplementary material for: Phosphorylation of luminal region of the SUN-domain protein Mps3 promotes nuclear envelope localization during meiosis
Source: eLife. 2021 Sep 29;10:e63119. doi: 10.7554/eLife.63119 (PMC8570693; doi:10.7554/eLife.63119)
Supplement: Supplementary file 1. [file elife-63119-supp1.docx]

**Supplementary File 1: Strain list**

| Strain Name | Genotype |
| --- | --- |
| NKY1551 | *MAT****a****/α*, *ho::LYS2/ho::LYS2*, *lys2/lys2*, *leu2::hisG/leu2::hisG*, *ura3/ura3*, *his4B-LEU2/his4X-LEU2-URA3*, *arg4-bgl/arg4-nsp* |
| MSY832/833 | *MAT****a****/α*, *ho::LYS2, ura3, leu2::hisG,* *trp1::hisG,* *lys2* |
| HKY404 | *MAT****a****/α*, *ho::LYS2/ho::LYS2*, *lys2/lys2*, *leu2::hisG/leu2::hisG*, *ura3/ura3*,*lys2/lys2,MPS3-3FLAG::KanMX6/ MPS3-3FLAG::KanMX6,NDJ1-3HA::KanMX6/NDJ1-3HA::KanMX6* |
| *PRY13* | *MAT****a****/α*, *ho::LYS2/ho::LYS2*, *lys2/lys2*, *leu2::hisG/leu2::hisG*, *ura3/ura3*, *MPS3-3FLAG::KanMX6/ MPS3-3FLAG::KanMX6, NDJ1-3HA::KanMX6/NDJ1-3HA::KanMX6,*  *cdc28-as1/cdc28-as1* |
| PRY72 | *MAT****a****/α*, *ho::LYS2/ho::LYS2*, *lys2/lys2*, *leu2::hisG/leu2::hisG*, *ura3/ura3*, *MPS3-3FLAG::KanMX6/MPS3-3FLAG::KanMX6, NDJ1-3HA::KanMX6/NDJ1-3HA::KanMX6,*  *cdc7-as3-9MYC/cdc7-as3-9MYC* |
| PRY 64 | *MAT****a****/α*, *ho::LYS2/ho::LYS2*, *lys2/lys2*, *leu2::hisG/leu2::hisG*, *ura3/ura3*, *MPS3-3GFP::KanMX6/MPS3-3GFP::KanMX6* |
| PRY71 | *MAT****a****/α*, *ho::LYS2/ho::LYS2*, *lys2/lys2*, *leu2::hisG/leu2::hisG*, *ura3/ura3*,*MPS3-3GFP::KanMX6/MPS3-3GFP::KanMX6, cdc28-as1/cdc28-as1* |
| PRY260 | *MAT****a****/α*, *ho::LYS2/ho::LYS2*, *lys2/lys2*, *leu2::hisG/leu2::hisG*, *ura3/ura3*, *MPS3-3GFP::KanMX6/MPS3-3GFP::KanMX6, cdc7-as3-9MYC/cdc7-as3-9MYC* |
| PRY115 | *MAT****a****/α*, *ho::LYS2/ho::LYS2*, *lys2/lys2*, *leu2::hisG/leu2::hisG*, *ura3/ura3*, *MPS3-3GFP::KanMX6/MPS3-3GFP::KanMX6, cdc7::KanMX6/cdc7::KanMX6, bob1-1/bob1-1* |
| PRY122 | *MAT****a****/α*, *ho::LYS2/ho::LYS2*, *lys2/lys2*, *leu2::hisG/leu2::hisG*, *ura3/ura3*, *mps3-AAA(T188A S189A S190A-PSTI)/ mps3-AAA (T188A S189A S190A-PSTI)* |
| PRY186 | *MAT****a****/α*, *ho::LYS2/ho::LYS2*, *lys2/lys2*, *leu2::hisG/leu2::hisG*, *ura3/ura3*, *mps3-AAA(T188A S189A S190A-PSTI)- 3GFP::KanMX6/mps3-AAA (T188A S189A S190A-PSTI) - 3GFP::KanMX6* |
| PRY163 | *MAT****a****/α*, *ho::LYS2/ho::LYS2*, *lys2/lys2*, *leu2::hisG/leu2::hisG*, *ura3/ura3*, *mps3-3A(T188A S189A S190A-PSTI)-3FLAG::KanMX6/ mps3-3A(T188A S189A S190A-PSTI)-3FLAG::KanMX6 ,NDJ1-3HA ::KanMX6/ NDJ1-3HA::KanMX6* |
| PRY138 | *MAT****a****/α*, *ho::LYS2/ho::LYS2*, *lys2/lys2*, *leu2::hisG/leu2::hisG*, *ura3/ura3*, *mps3-AAA(T188A S189A S190A-PSTI)/ mps3-AAA (T188A S189A S190A-PSTI), Rap1-GFP::LEU2/Rap1-GFP::LEU2* |
| PRY201 | *MAT****a****/α*, *ho::LYS2/ho::LYS2*, *lys2/lys2*, *leu2::hisG/leu2::hisG*, *ura3/ura3*, *MPS3-DDD(T188D S189D S190D–BSPEI)/MPS3-DDD(T188D S189D S190D–BSPEI)* |
| PRY211 | *MAT****a****/α*, *ho::LYS2/ho::LYS2*, *lys2/lys2*, *leu2::hisG/leu2::hisG*, *ura3/ura3*, *MPS3-DDD(T188D S189D S190D–BSPEI)-3GFP::KanMX6/ MPS3-DDD(T188D S189D S190D–BSPEI)-3GFP::KanMX6* |
| PRY219 | *MAT****a****/α*, *ho::LYS2/ho::LYS2*, *lys2/lys2*, *leu2::hisG/leu2::hisG*, *ura3/ura3*, *MPS3-DDD(T188D S189D S190D–BSPEI)-3FLAG::KanMX6/ MPS3-DDD(1 T188D S189D S190D–BSPEI)-3FLAG::KanMX6, NDJ1-3HA ::KanMX6/NDJ1-3HA::KanMX6* |
| PRY236 | *MAT****a****/α*, *ho::LYS2/ho::LYS2*, *lys2/lys2*, *leu2::hisG/leu2::hisG*, *ura3/ura3*, *MPS3-DDD(T188D S189D S190D–BSPEI)/MPS3-DDD(T188D S189D S190D–BSPEI), Rap1-GFP::LEU2/Rap1-GFP::LEU2* |
| PRY301 | *MAT****a****/α*, *ho::LYS2/ho::LYS2*, *lys2/lys2*, *leu2::hisG/leu2::hisG*, *ura3/ura3*, *MPS3-DDD(T188D S189D S190D–BSPEI)-3GFP::KanMX6/ MPS3-DDD(T188D S189D S190D–BSPEI)-3GFP::KanMX6, cdc28-as1/cdc28-as1* |
| PRY272 | *MAT****a****/α*, *ho::LYS2/ho::LYS2*, *lys2/lys2*, *leu2::hisG/leu2::hisG*, *ura3/ura3*, *MPS3-DDD(T188D S189D S190D–BSPEI)-3GFP::KanMX6/ MPS3-DDD(T188D S189D S190D–BSPEI)-3GFP::KanMX6, cdc7-as3::9MYC/cdc7-as3::9MYC* |
| HKY167 | *MAT****a****/α*, *ho::LYS2/ho::LYS2*, *lys2/lys2*, *leu2::hisG/leu2::hisG*, *ura3/ura3*, *RAP1-GFP::LEU2/RAP1-GFP::LEU2* |
| PRY68 | *MAT****a****/α*, *ho::LYS2/ho::LYS2*, *lys2/lys2*, *leu2::hisG/leu2::hisG*, *ura3/ura3*, *RAP1-GFP::LEU2/RAP1-GFP::LEU2, cdc28-as1/cdc28-as1* |
| PRY79 | *MAT****a****/α*, *ho::LYS2/ho::LYS2*, *lys2/lys2*, *leu2::hisG/leu2::hisG*, *ura3/ura3*, *RAP1-GFP::LEU2/RAP1-GFP::LEU2, cdc7-as3-9MYC/cdc7-as3-9MYC* |
| PRY116 | *MAT****a****/α*, *ho::LYS2/ho::LYS2*, *lys2/lys2*, *leu2::hisG/leu2::hisG*, *ura3/ura3*, *RAP1-GFP::LEU2/RAP1-GFP::LEU2, cdc7::KanMX6/ cdc7::KanMX6, bob1-1/bob1-1* |
| PRY303 | *MAT****a****/α*, *ho::LYS2/ho::LYS2*, *lys2/lys2*, *leu2::hisG/leu2::hisG*, *ura3/ura3*, *MPS3-DDD(T188D S189D S190D–BSPEI)-3GFP::KanMX6/ MPS3-DDD(T188D S189D S190D–BSPEI)-3GFP::KanMX6, Rap1-GFP::LEU2/Rap1-GFP::LEU2, cdc28-as1/cdc28-as1* |
| PRY309 | *MAT****a****/α*, *ho::LYS2/ho::LYS2*, *lys2/lys2*, *leu2::hisG/leu2::hisG*, *ura3/ura3*, *MPS3-DDD(T188D S189D S190D–BSPEI)-3GFP::KanMX6/ MPS3-DDD(T188D S189D S190D–BSPEI)-3GFP::KanMX6, Rap1-GFP::LEU2/Rap1-GFP::LEU2, cdc7-as3::9MYC/cdc7-as3::9MYC* |
| PRY322 | *MAT****a****/α*, *ho::LYS2/ho::LYS2*, *lys2/lys2*, *leu2::hisG/leu2::hisG*, *ura3/ura3*, *MPS3-DDD(T188D S189D S190D–BSPEI)-3GFP::KanMX6/ MPS3-DDD(T188D S189D S190D–BSPEI)-3GFP::KanMX6, Rap1-GFP::LEU2/Rap1-GFP::LEU2, cdc7::KanMX6/ cdc7::KanMX6, bob1-1/bob1-1* |
| PRY192 | *MAT****a****/α*, *ho::LYS2/ho::LYS2*, *lys2/lys2*, *leu2::hisG/leu2::hisG*, *ura3/ura3*, *MPS3-3GFP::KanMX6/MPS3-3GFP::KanMX6 ndj1::KanMX6/ndj1::KanMX6* |
| PRY198 | *MAT****a****/α, ho::LYS2/ho::LYS2*, *lys2/lys2*, *leu2::hisG/leu2::hisG*, *ura3/ura3*, *MPS3-3GFP::KanMX6/MPS3-3GFP::KanMX6, csm4::KanMX6/csm4::KanMX6* |
| PRY514 | *MAT****a****/α*, *ho::LYS2/ho::LYS2*, *lys2/lys2*, *leu2::hisG/leu2::hisG*, *ura3/ura3*, *Mps3-MER2S (swap)-SacI-Flag/ Mps3-MER2S (swap)-SacI-Flag, Rap1-GFP::LEU2/Rap1-GFP::LEU2, NDJ1-HA/NDJ1-HA* |
| PRY518 | *MAT****a****/α*, *ho::LYS2/ho::LYS2*, *lys2/lys2*, *leu2::hisG/leu2::hisG*, *ura3/ura3*, *Mps3-MER2S (swap)-SacI-Flag/ Mps3-MER2S (swap)-SacI-Flag, Rap1-GFP::LEU2/Rap1-GFP::LEU2, Ndj1-HA/ Ndj1-HA* |
| KSY220/221 | *MAT****a****/α*, *ho::LYS2/ho::LYS2*, *lys2/lys2*, *leu2::hisG/leu2::hisG*, *ura3/ura3*, *mps3-S190A (-PSTI)-3GFP::KanMX6/mps3-S190A(-PSTI)-3GFP::KanMX6* |
| KSY407/409 | *MAT****a****/α*, *ho::LYS2/ho::LYS2*, *lys2/lys2*, *leu2::hisG/leu2::hisG*, *ura3/ura3*, *mps3-S189A-(BSPEI)-3GFP::KanMX6/mps3-S189A-(BSPEI)-3GFP::KanMX6* |
